# Supplementary material for: Achieving high-efficiency emission depletion nanoscopy by employing cross relaxation in upconversion nanoparticles
Source: Nat Commun. 2017 Oct 20;8:1058. doi: 10.1038/s41467-017-01141-y (PMC5648820; doi:10.1038/s41467-017-01141-y)
Supplement: Supplementary file 1 — Supplementary Information [file 41467_2017_1141_MOESM1_ESM.pdf]

Supplementary Methods

Materials and Reagents

Gadolinium(III) acetate hydrate (99.9%), yttrium(III) acetate hydrate (99.9%), ytterbium(III) acetate hydrate (99.9%), thulium(III) acetate hydrate (99.9%) and terbium(III) acetate hydrate (99.9%), diethylene glycol (DEG,98%), N-(3-Dimethylaminopropyl)-N'-ethylcarbodiimide hydrochloride (EDC, ≥98%), rhodamine were purchased from Sigma-Aldrich. Sodium hydroxide (NaOH, >98%), ammonium fluoride (NH_4F, >99.99%), 1-octadecene (90%) and oleic acid (90%) were purchased from Aladdin®, China. Methanol (reagent grade), ethanol (reagent grade), cyclohexane (reagent grade), hydrochloric acid (HCl, AR, 36%-38%) were purchased from Sinopharm Chemical Reagent Co., China. Poly(acrylic acid) (PAA, Mw~2000) was purchased from Shanghai Makclin Biochemical Co., Ltd. N-Hydroxysuccinimide (NHS, 98%), Dulbecco's Modified Eagle Medium (DMEM), fetal bovine serum (FBS), Phosphate-buffered Saline (PBS) and trypsin (0.25%) were purchased from Thermo Fisher Scientific. 2-(N-Morpholino)ethanesulfonic (MES) acid Buffer (0.1 mol/L, pH=6.0) were bought from Leagene Biotechnology co., Beijing. Paraformaldehyde (PFA, 4%）was purchased from Invitrogen. HeLa cells source was provided by Sun Yat-Sen University (Guangzhou, China). Triton X-100 was purchased from Amresco. Quick Block™ blocking buffer and Quick Block™ primary antibody dilution buffer for immunol staining were purchased from Beyotime, China. The primary antibody (anti-desmin monoclonal antibody, Y66, ab32362) was purchased from Abcam, Cambridge, UK, and the secondary antibody (Goat Anti-rabbit IgG, bs-0295G) was purchased from Bioss, China. Brilliant Blue G250 (AR) and Phosphoric acid (AR, ≥85 wt.% in H_2O) were purchased from Aladdin®, China. All the reagents were used as received without further purification.

Characterization methods

Transmission electron microscopy (TEM). TEM, high resolution TEM (HRTEM) images and selected area electron diffraction (SAED) patterns were obtained on a electron microscope (JEM-2100HR, JEOL). The size and shape of the nanoparticles were determined from TEM and HRTEM images, and the crystalline phase can be calculated from the SAED images.

Dynamic light scattering (DLS). DLS characterization for the hydrodynamic size of UCNPs were performed using a NanoPlus granulometer (Micromeritics instrument Ltd.). The sample with a concentration of 0.1 mg/mL was used in the measurement.

Fourier transform infrared spectroscopy. The functional groups on the surface of nanoparticles were characterized by Fourier transform infrared (FTIR) spectroscopy using a spectrometer (Nicolet 6700, Thermo Scientific).

The Bradford protein assay. The Bradford protein assay was used to evaluate the secondary antibody (IgG) in order to make sure they were covalently linked to the UCNPs[1]. Firstly, the protein reagent was prepared as described below. Coomassie Brilliant Blue G-250 (10 mg) was dissolved into 5 mL ethanol (95%). Then 10 mL 85% (w/v) phosphoric acid was added into the mixture. The resulting solution was further diluted to 100 mL by adding deionized (DI) water. Final concentrations in the prepared protein reagent were 0.01% (w/v) Coomassie Brilliant Blue G-250, 4.7% (w/v) ethanol, and 8.5% (w/v) phosphoric acid. In the measurement, 900 μL protein reagent (prepared with Coomassie Brilliant Blue G-250) and 100 μL sample solution (IgG, IgG-UCNPs) were mixed in a thin cuvette and the absorption of the mixture was detected using a UV-VIS spectrometer (Perkin Elmer-Lambda 950) with protein reagent/PBS (v/v=9:1) solution as the reference sample.

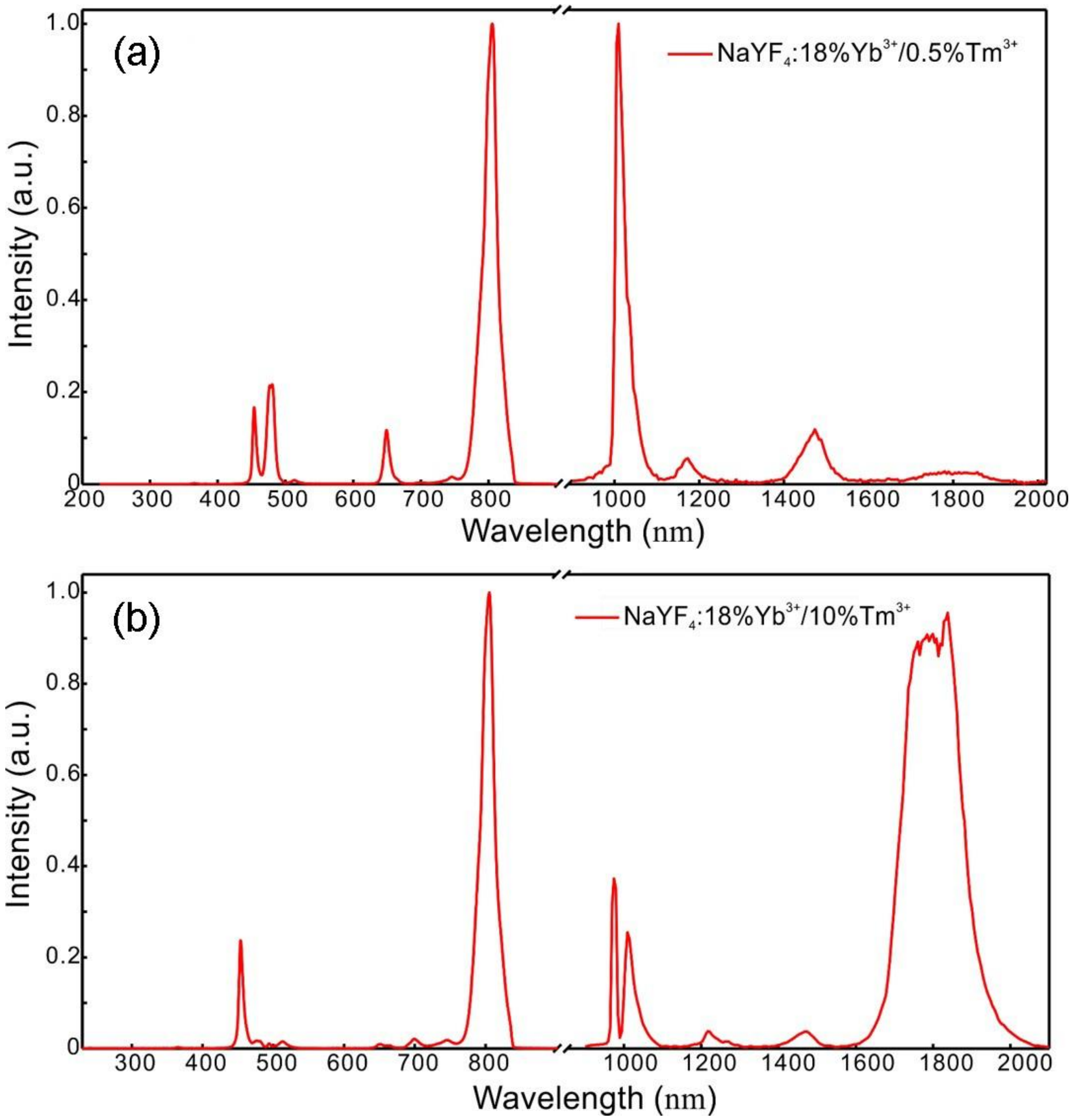

Supplementary Figure 1 | Upconversion/downconversion full emission spectra of the as-prepared Tm^{3+}-doped nanoparticles under 975-nm laser excitation. (a) $NaYF_4$:18%Yb^{3+},0.5%Tm^{3+} nanoparticles; **(b)** $NaYF_4$:18%Yb^{3+},10%Tm^{3+} nanoparticles. The spectra were individually normalized in each spectral range, including 240-900 nm (collected by a visible light Spectrometer), 900-2100 nm (collected by an NIR Spectrometer), separated by a cutoff mark. The 975-nm laser power density was 700 kW cm^{-2}.

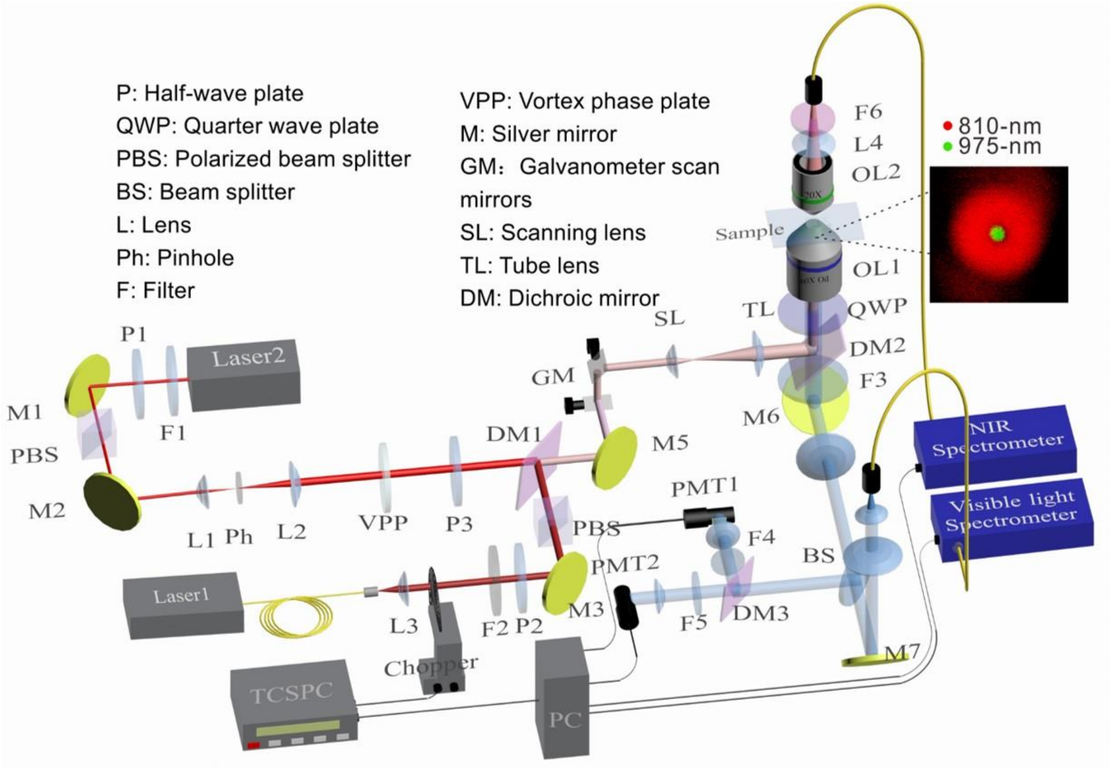

Supplementary Figure 2 | Scheme of the lab-built optical measurement setup. F1: 810-nm band-pass Filter; F2: 975-nm band-pass Filter; F3: 775-nm short-pass Filter; F4: 455-nm band-pass Filter; F5: 550-nm band-pass Filter; F6: 980-nm long-pass filter; L1: Lens focus 50 mm; L2: lens focus 100 mm; L3, L4: Lens focus 25 mm; Ph: 25 μm Pinhole; P1, P2, P3: half-wave plates; QWP: Quarter-wave plate; VPP: vortex phase plate; GM: Galvanometer scanning mirrors; DM1: 950-nm short-pass dichroic mirror; DM2: 775-nm short-pass dichroic mirror; DM3: 485-nm long-pass dichroic mirror; SL: Scanning lens; OL1: 60× objective lens; OL2: 20× objective lens; PMT1, PMT2: photomultiplier tubes; M1-M7: sliver reflection mirrors.

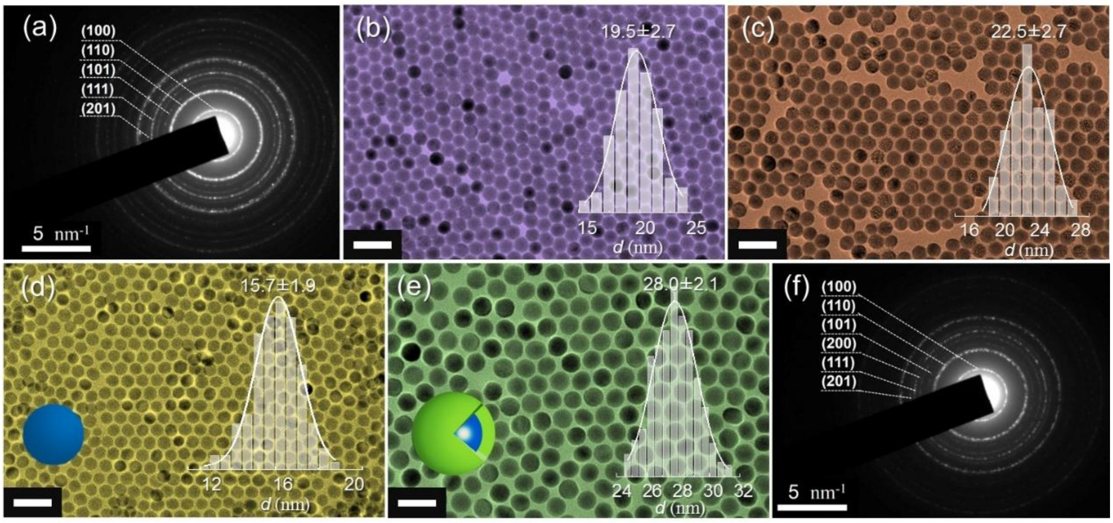

Supplementary Figure 3 | Transmission electron microscopy (TEM) images and selected area electron diffraction (SAED) patterns of the as-synthesized UCNPs. **(a)** The SAED pattern of the $NaYF_4$:18%Yb^{3+},10%Tm^{3+} used for optical depletion and super-resolution imaging, matching a hexagonal $NaYF_4$ lattice (JCPDS file number 16-0334). **(b)** TEM image of the as-prepared low Tm^{3+}-doped $NaYF_4$:18%Yb^{3+},0.5%Tm^{3+} UCNPs, average size: 19.5±2.7 nm in diameter. **(c)** TEM image of the as-prepared highly Tm^{3+}-doped $NaYF_4$:18%Yb^{3+},20%Tm^{3+} UCNPs, average size: 22.5±2.7 nm in diameter. **(d)** TEM image of the as-prepared core $NaGdF_4$:40%Yb^{3+},10%Tm^{3+}, average size: 15.7±1.9 nm in diameter. **(e)** TEM image of the as-prepared core-shell $NaGdF_4$:40%Yb^{3+},10%Tm^{3+}@$NaGdF_4$:15%Tb^{3+} UCNPs, average size: 28.0±2.1 nm in diameter. **(f)** The SAED pattern of the as-prepared core-shell $NaGdF_4$:40%Yb^{3+}, 10%Tm^{3+}@$NaGdF_4$:15%Tb^{3+}, matching a hexagonal $NaGdF_4$ lattice (JCPDS file number 27-0699). The scale bars in TEM images are 50 nm.

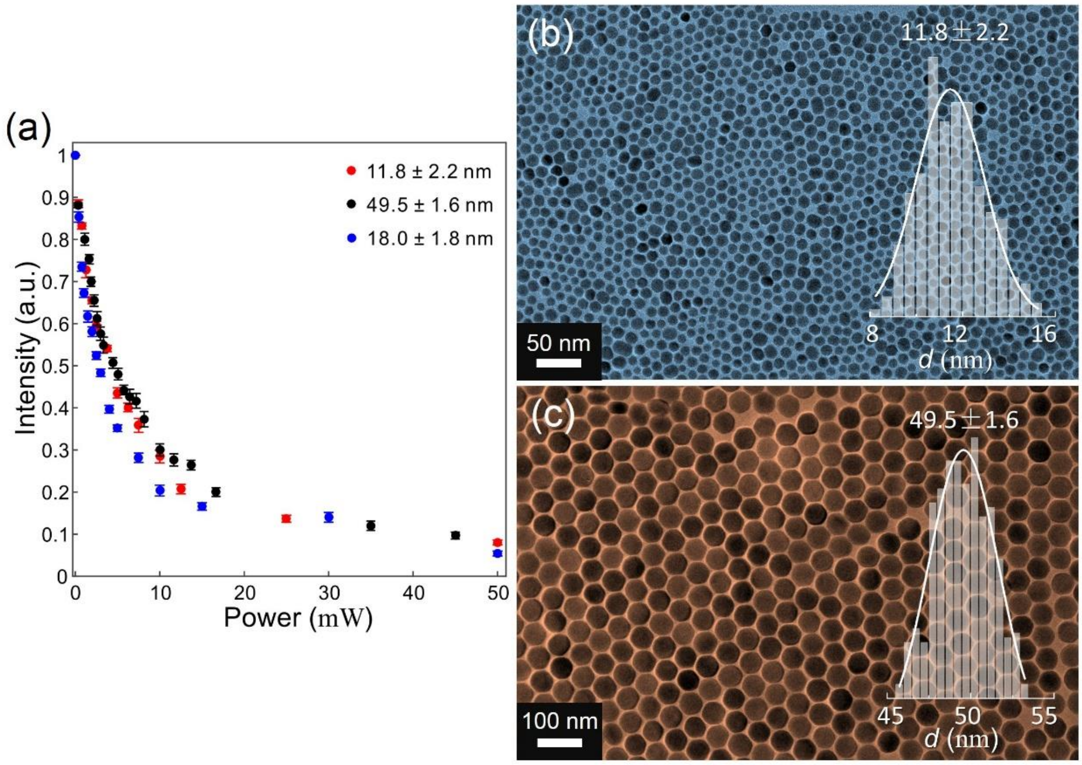

Supplementary Figure 4 | The effect of nanoparticle size on the depletion efficiency. (a) The depletion efficiencies of three different sized UCNP samples at different powers of the 810-nm depletion laser. **(b)** TEM image of the high Tm^{3+}-doped $NaGdF_4$:18%Yb^{3+}, 10%Tm^{3+} UCNPs, average size: 11.8±2.2 nm in diameter. This is the small nanoparticles employed in the immunolabelling cytoskeleton experiments. **(c)** TEM image of the high Tm^{3+}-doped $NaYF_4$:18%Yb^{3+}, 10%Tm^{3+} UCNPs, average size: 49.5±1.6 nm in diameter. The 455-nm emission of all the samples can be efficiently depleted by the 810-nm laser with slight difference in the depletion efficiency, showing insignificant size effect. The TEM image of the measured $NaYF_4$:18%Yb^{3+}, 10%Tm^{3+} with an average diameter of 18.0±1.8 nm was shown in Figure 1a of the main text. Error bars represent ±1s.d.

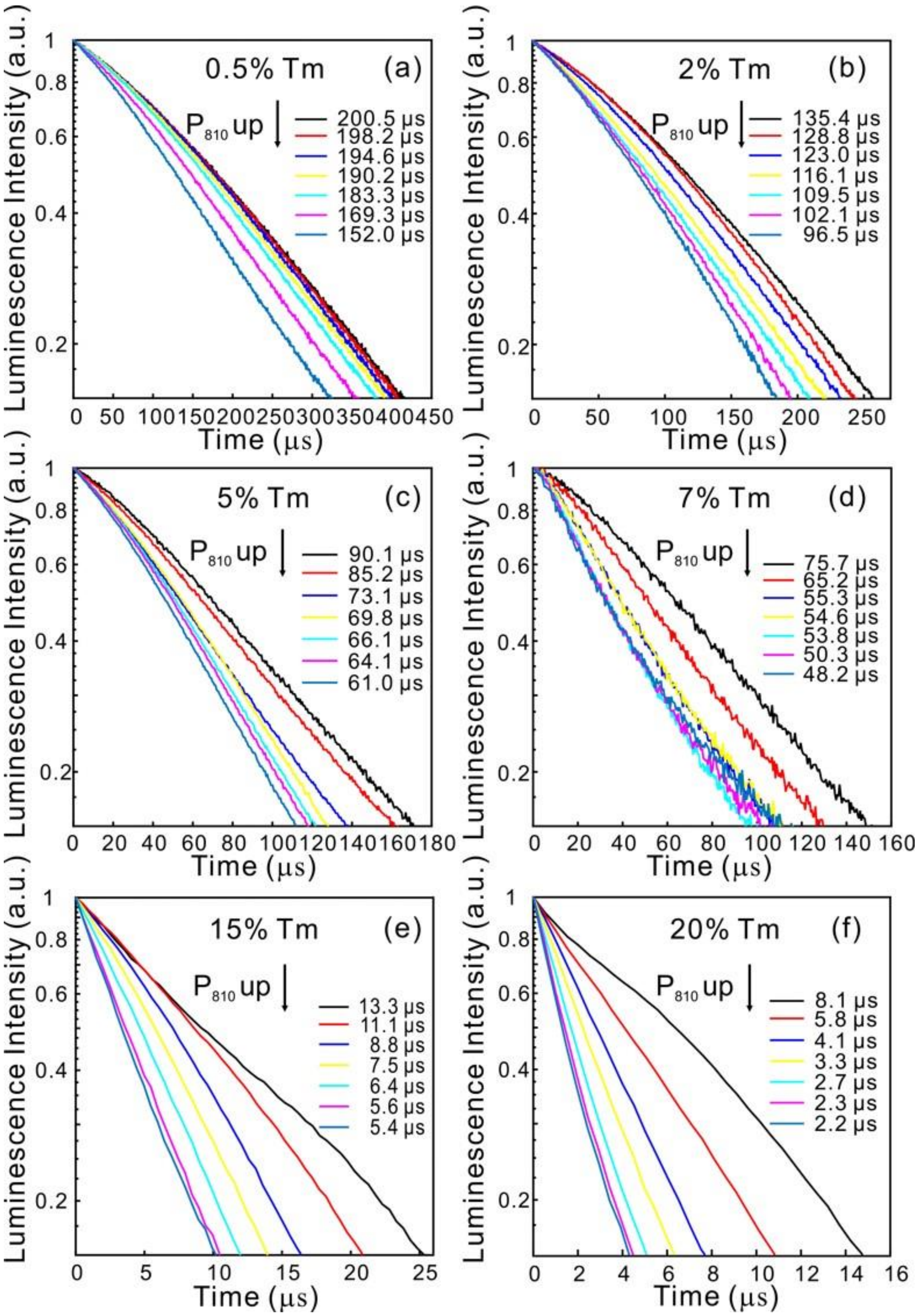

Supplementary Figure 5 | The 810-nm laser intensity dependent lifetime decrease of the 455-nm emission of UCNPs. Samples of different Tm^{3+} doping concentrations, **(a)** 0.5%Tm^{3+}, **(b)** 2% Tm^{3+}, **(c)** 5% Tm^{3+}, **(d)** 7% Tm^{3+}**, (e)** 15%

Tm^{3+}, **(f)** 20% Tm^{3+}, were measured under 975-nm&810-nm simultaneous excitation. The 975-nm laser beam was mechanically modulated using a chopper, while the 810-nm laser beam irradiated the sample continuously. The power density of the 810-nm depletion laser beam at the sample was adjusted to 0 MW cm^{-2}, 0.3 MW cm^{-2}, 1.48 MW cm^{-2}, 2.95 MW cm^{-2}, 5.9 MW cm^{-2}, 11.8 MW cm^{-2}, and 15.7 MW cm^{-2} in sequence, and an emission decay curve was recorded at each power density. The lifetime of the 455-nm luminescence decreased with increasing the power of the 810-nm depletion laser in all the measured UCNPs, indicating the occurrence of the stimulated emission process $^1D_2 \xrightarrow{810\ nm} {}^3F_2$. In principle, the lifetime of stimulated emission is determined by the product of the stimulated emission cross-section (σ_{STED}) and the photon flux (ρ_{STED}) of the depletion laser by $\tau_{STED} = 1/k_{STED} = 1/(\sigma_{STED} \times \rho_{STED})$. According to Ref. [2], STED reduces the lifetime of the excited state from $\tau_f = 1/k_{fl}$ to $\tau = 1/(k_{fl} + \sigma_{STED} \times \rho_{STED}) = 1/(k_{fl} + \sigma_{STED} \times I_{STED} \times \lambda_{STED}/(hc))$, where h is the Planck's constant, and c is the speed of light. Taking I_{STED} = 15.7 MW cm^{-2} and $\sigma_{STED} = 10^{-21}$-10^{-20} cm^2 [3, 4, 5, 6], it yields $\tau_{STED} \approx$ 1.5-6.7 μs, which is three orders of magnitude longer than the stimulated emission lifetime in organic fluorescent dyes (ns versus ps).

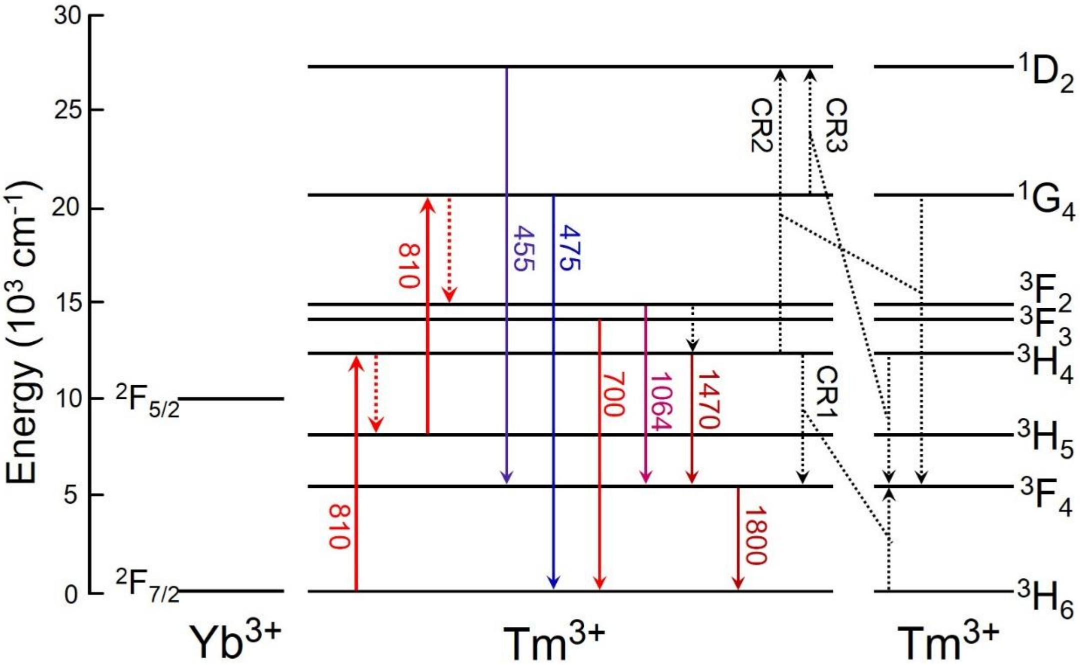

Supplementary Figure 6 | Upconversion mechanism in Yb^{3+}/Tm^{3+} co-doped UCNPs under single 810-nm excitation. When excited by an 810-nm CW laser, electrons at the ground state $^3H_6$ are firstly excited to the $^3H_4$ state through a ground state absorption (GSA) process, and then decay to the $^3H_5$ state via a multiphonon relaxation (MPR) process. The following excited state absorption (ESA) process drives the electrons at the $^3H_5$ state to the $^1G_4$ state, which is responsible for the 475-nm blue emission. The 1064-nm and 700-nm emissions are attributed to the $^3F_{2,3}$ state, populated from the $^1G_4$ state through a MPR process. The $^1D_2$ state is primarily populated by cross relaxation with two routes (CR2 and CR3), namely, $^3H_4 + {}^1G_4 \rightarrow {}^1D_2 + {}^3F_4$ and $^1G_4 + {}^3H_4 \rightarrow {}^1D_2 + {}^3F_4$. The ESA process: $^3F_2 \rightarrow {}^1D_2$ is an alternative pathway to populate the $^1D_2$ state. However, under the condition of single 810-nm excitation, the contribution of this ESA process is small, as the $^3F_2$ state remains only a small population[7].

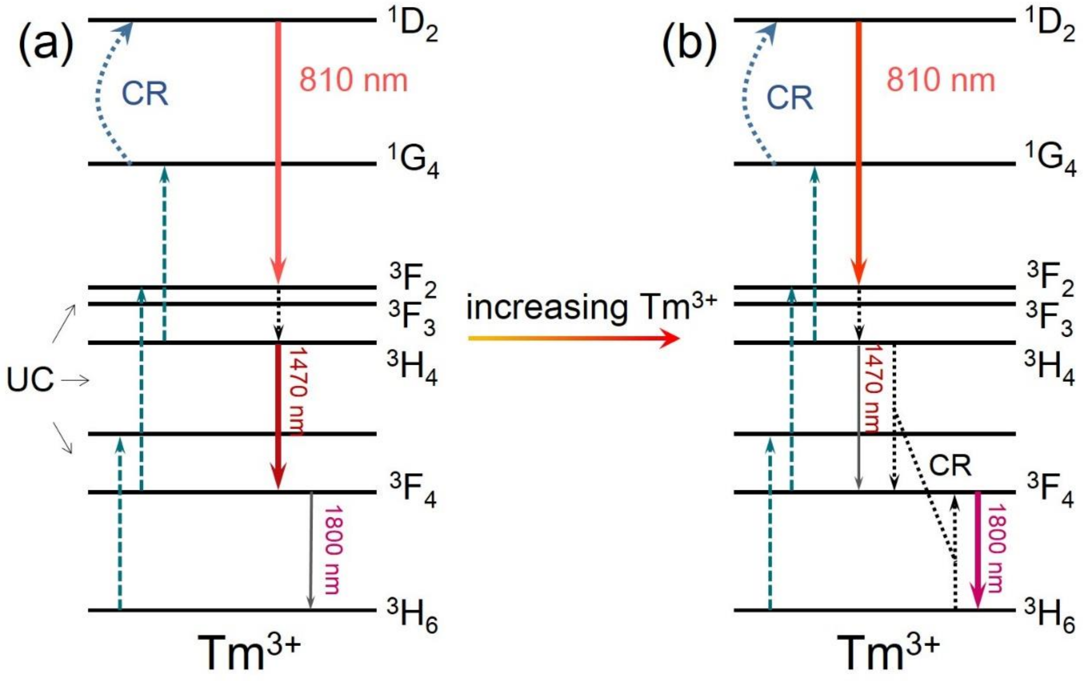

Supplementary Figure 7 | The luminescent mechanism of 1470-nm/1800-nm emissions from $NaYF_4$:18%Yb^{3+}, x%Tm^{3+} UCNPs with varying Tm^{3+} concentrations. Simplified energy level diagrams of **(a)** $NaYF_4$:18%Yb^{3+},0.5%Tm^{3+} and **(b)** $NaYF_4$:18%Yb^{3+},10%Tm^{3+}. The 1470-nm emission is generated from $^3H_4$ and 1800-nm emission is generated from $^3F_4$. With the increase of Tm^{3+} concentration, the 1470-nm emission intensity decreases while the 1800-nm emission intensity increases. The intensity ratio between 1470 nm and 1800 nm I_{1470}/I_{1800} changed from 5 to 1/32 (Fig. 2e in the main text). The main reason for these intensity variations is the interionic cross relaxation: $^3H_4 + {}^3H_6 \rightarrow {}^3F_4 + {}^3F_4$ between Tm^{3+} ions in high Tm^{3+}-doped UCNPs[8, 9], which directs the electrons at $^3H_4$ to $^3F_4$.

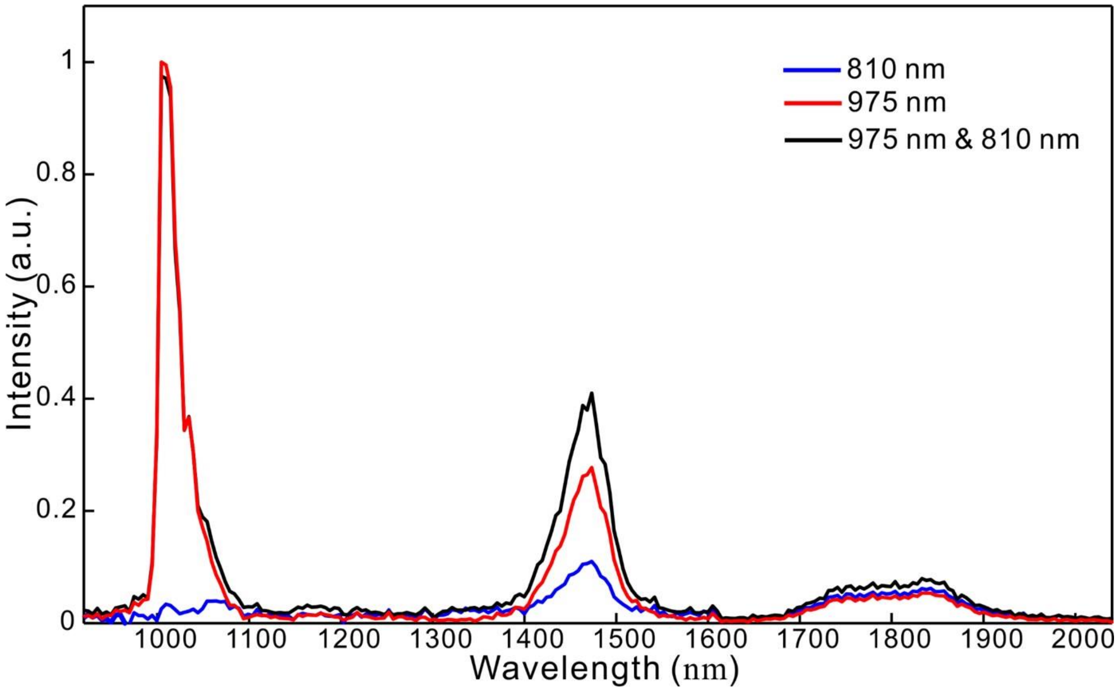

Supplementary Figure 8 | NIR emission spectra of low Tm^{3+}-doped UCNPs ($NaYF_4$:18%Yb^{3+},0.5%Tm^{3+}) under different excitation conditions. The power density is 700 kW cm^{-2} for the 975-nm CW laser beam, and 17.7 MW cm^{-2} for the 810-nm CW laser beam. Note that the intensities of 1470-nm and 1800-nm emissions were both increased with the addition of the 810-nm laser.

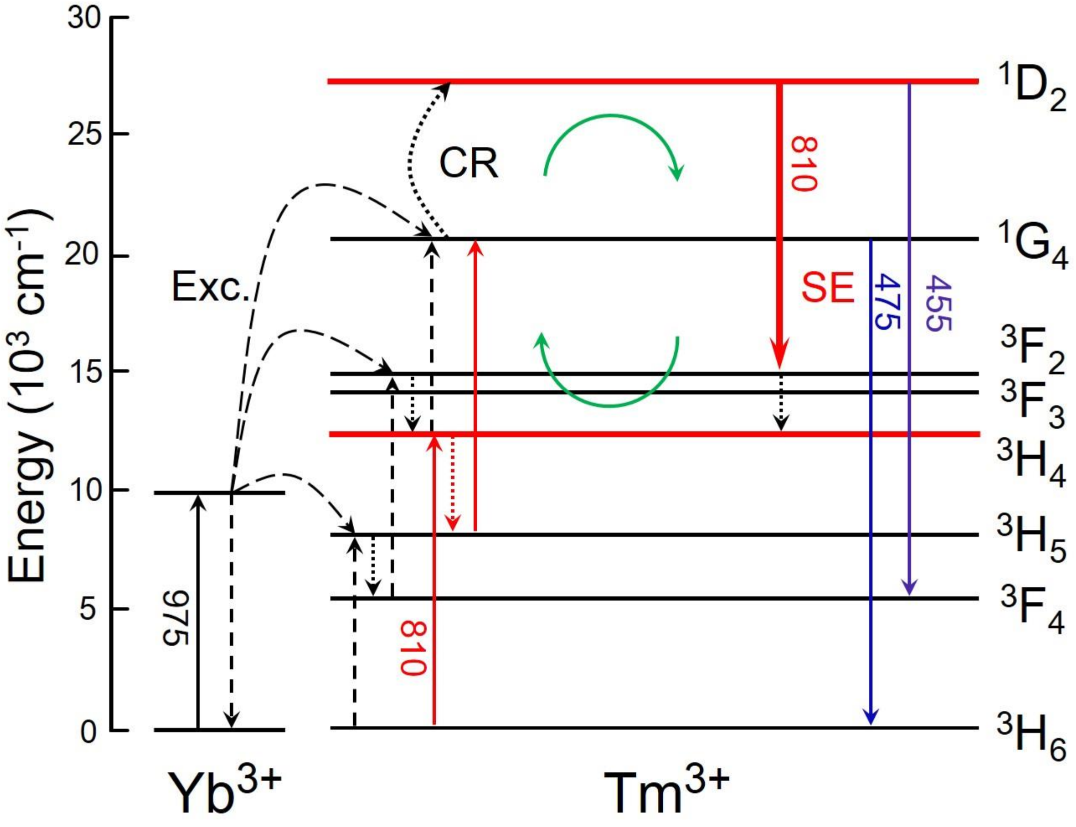

Supplementary Figure 9 | An electron recycling pathway caused by the 975-nm laser in low Tm^{3+}-doped $NaYF_4$:Yb^{3+},Tm^{3+} UCNPs. The electrons at the $^1D_2$ state can be transferred to the $^3F_2$ state by the depletion laser at 810 nm and then quickly decay to the $^3H_4$ state nonradiatively. The electrons at the $^3H_4$ state would be excited by the 975-nm excitation laser to populate the $^1D_2$ state (denoted by an electron cycling pathway marked by green curved arrows), compromising the depletion effect of the 810-nm beam.

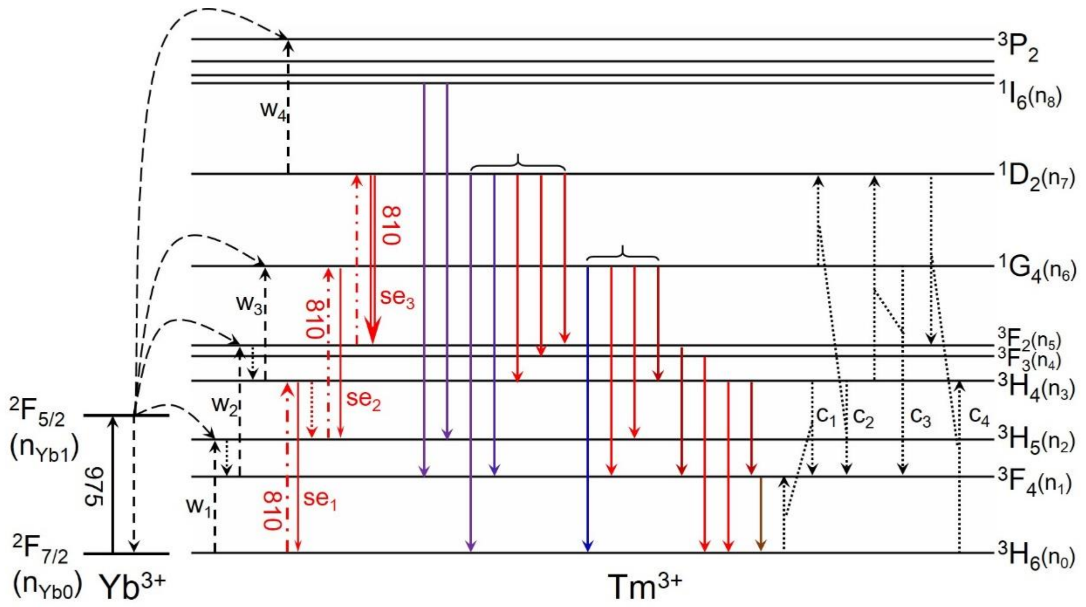

Supplementary Figure 10 | The energy diagram used in the modelling and simulation of the 455-nm luminescence depletion of $NaYF_4$:18%Yb^{3+}, 10%Tm^{3+} and $NaYF_4$:18%Yb^{3+}, 0.5%Tm^{3+}.

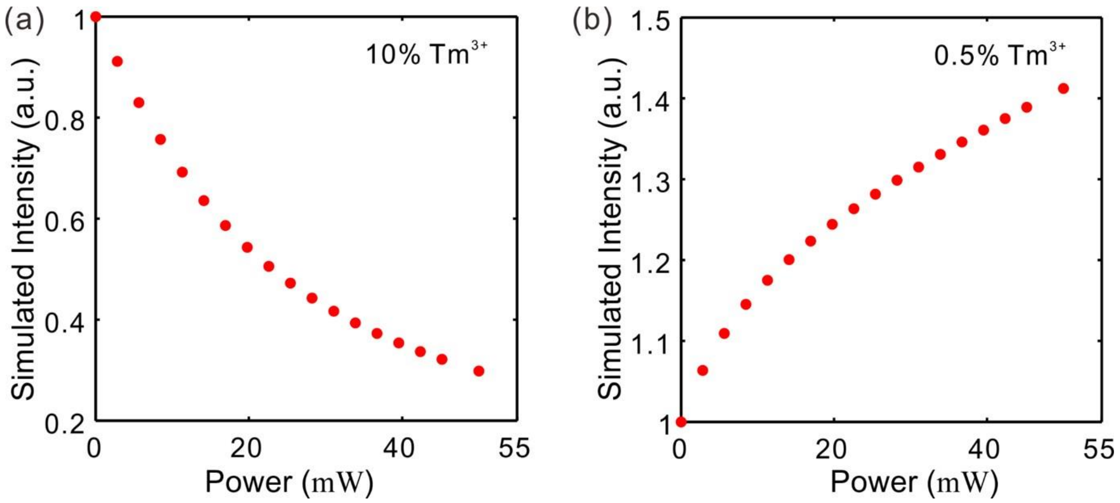

Supplementary Figure 11 | Simulated dependence of the intensity change of the 455-nm luminescence on the power of the 810-nm depletion beam. (a) High Tm^{3+}-doped UCNPs (10%). **(b)** Low Tm^{3+}-doped UCNPs (0.5%).

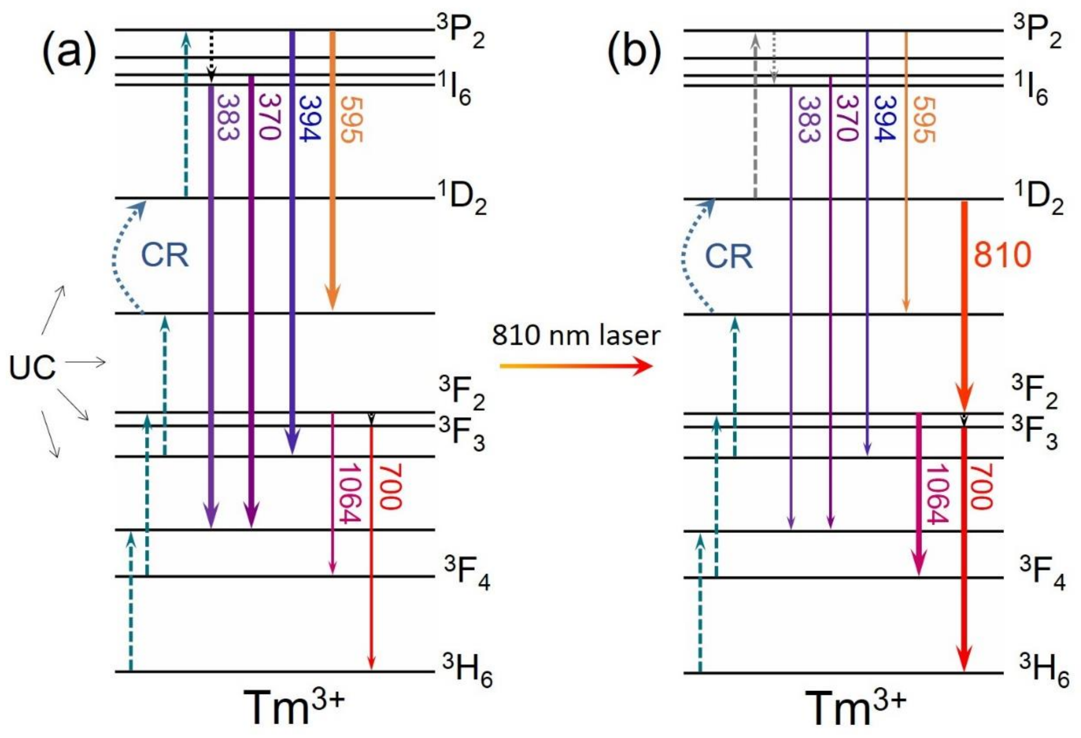

Supplementary Figure 12 | The luminescence mechanism of the 383-nm/394-nm/ 595-nm emissions and 700-nm/1064-nm emissions from $NaYF_4$:18%Yb^{3+}, 10%Tm^{3+} UCNPs under 975-nm excitation. (a) Without 810-nm irradiation. **(b)**With 810-nm irradiation. The 383-nm emission is generated from $^1I_6$, and the 595-nm, 394-nm emissions are generated from $^3P_2$, and the 1064-nm and 700-nm emissions are generated from $^3F_2$ and $^3F_3$ respectively. Energy levels $^1I_6$ and $^3P_2$ are populated from $^1D_2$ through ETU between Yb^{3+} and Tm^{3+} ions[10].

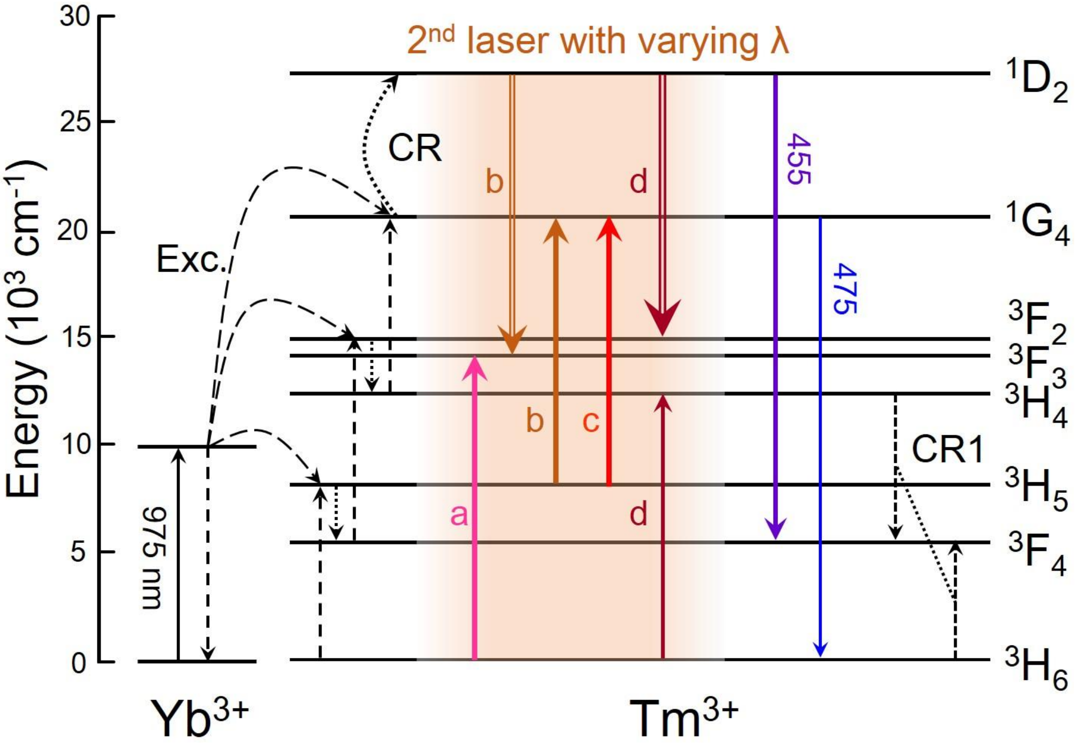

Supplementary Figure 13 | Proposed energy level mechanism for the depletion-laser-wavelength (700-815 nm) dependent depletion/enhancement effect on the 455-nm emission of $NaYF_4$:18%Yb^{3+}, 10%Tm^{3+} UCNPs. (a) The laser wavelengths in the range of 700-730 nm (indicated by letter "a") can match the GSA process of the $^3H_6 \rightarrow {}^3F_3$ transition[11, 12]; **(b)** The laser wavelengths in the range of 720-770 nm (indicated by letter "b") can match both the emission spectrum of the $^1D_2 \rightarrow {}^3F_3$ transition (centered at around 745 nm) and ESA spectrum of the $^3H_5 \rightarrow {}^1G_4$ transition[13, 14]. **(c)** The laser wavelengths in the range of 730-790 nm (indicated by letter "c") can match the ESA spectrum of the $^3H_5 \rightarrow {}^1G_4$ transition (centered at around 765 nm)[15]; **(d)** The laser wavelengths in the range of 770-815 nm (indicated by letter "d") can match both the GSA spectrum of the $^3H_6 \rightarrow {}^3H_4$ transition (centered at around 780 nm) and the emission spectrum of the $^1D_2 \rightarrow {}^3F_2$ transition[15].

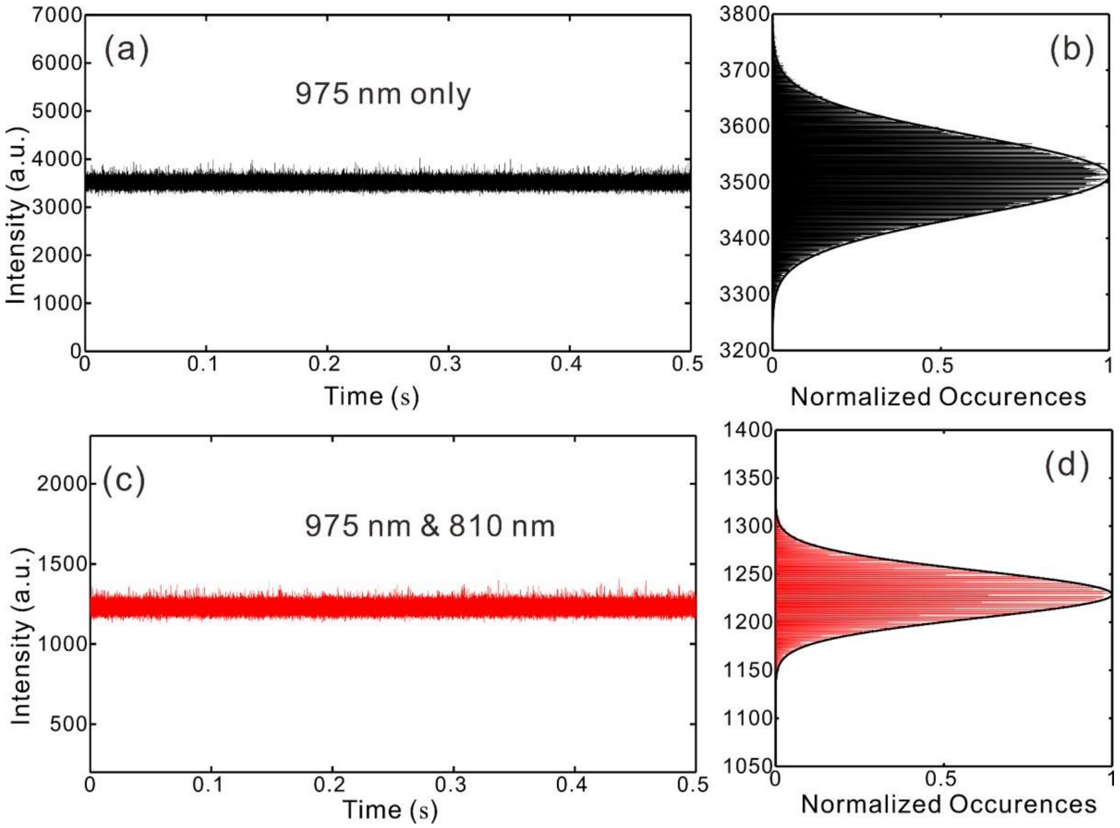

Supplementary Figure 14 | Nonblinking behavior of UCNPs under single 975-nm excitation and 975-nm&810-nm co-irradiation. Time trace recording for 0.5 s under **(a)** single 975-nm excitation (power density 700 kW cm^{-2}) and **(c)** 975-nm&810-nm simultaneous excitation with depletion efficiency of about 65%. The temporal resolution for each data point is 2 μs. The zoom-in time trace and histogram of emission intensity exhibit no on/off blinking behavior, as shown in **(b)** and **(d)**. According to the number of data point, the histogram of experimental data is well fitted with a normal distribution function for the case of single 975-nm excitation and 975-nm&810-nm co-irradiation.

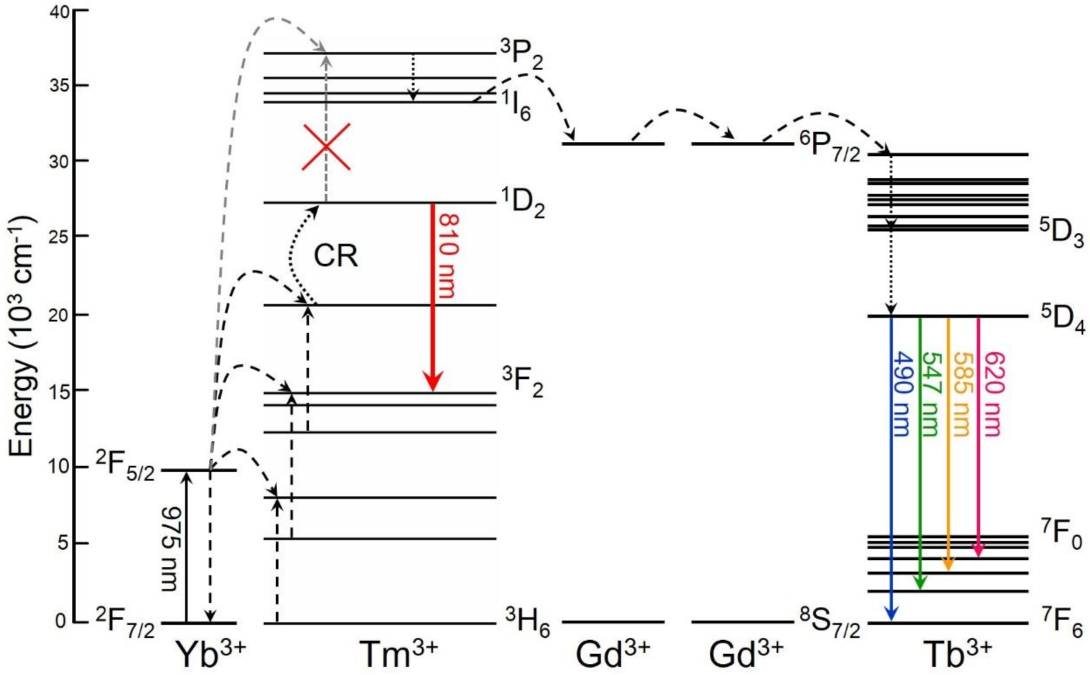

Supplementary Figure 15 | Luminescence depletion mechanism of Tb^{3+} ions in core-shell $NaGdF_4$:40%Yb^{3+}, 10%Tm^{3+}@$NaGdF_4$:15%Tb^{3+} UCNPs. Under single 975-nm excitation, the Tm^{3+} ions at the $^1I_6$ state transferred energy to Tb^{3+} ions through Gd^{3+} sublattice and populate the $^6P_{7/2}$ state of Tb^{3+} ions[16]. After a series of nonradiative relaxations, Tb^{3+} emits 490-nm/547-nm/585-nm/620-nm luminescence from the $^5D_4$ state. With the addition of the 810-nm depletion laser, the population of the $^1D_2$ state of Tm^{3+} was depleted, inhibiting further upconversion to upper energy levels including $^1I_6$. The depopulation of the $^1I_6$ state of Tm^{3+} caused the luminescence decrease of Tb^{3+} ions.

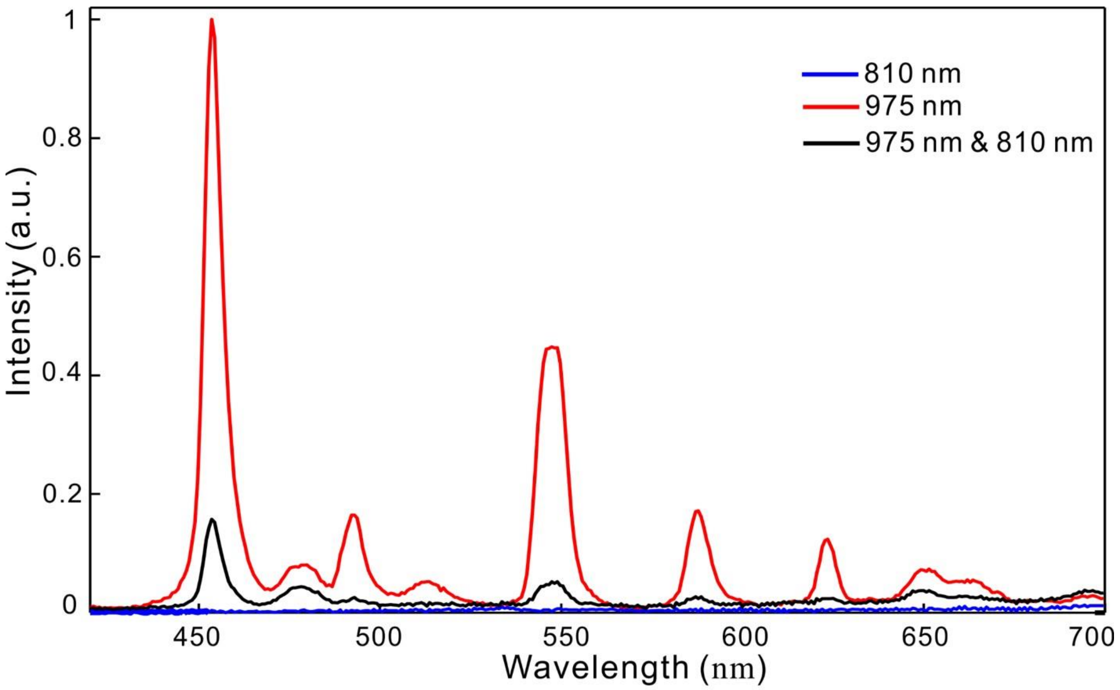

Supplementary Figure 16 | Photoluminescence of $NaGdF_4$:40%Yb^{3+}, 10%Tm^{3+}@$NaGdF_4$:15%Tb^{3+} UCNPs under different irradiation conditions. The photoluminescence spectra of $NaGdF_4$:40%Yb^{3+}, 10%Tm^{3+}@$NaGdF_4$:15%Tb^{3+} under single 975-nm CW laser irradiation (red solid line, power density 700 kW cm^{-2}), single 810-nm CW laser irradiation (blue solid line, power density 17.7 MW cm^{-2}), and 975-nm&810-nm simultaneous irradiation (black solid line). The 490-nm/547-nm/585-nm/620-nm upconversion emission were emitted from Tb^{3+} pumped via Gd^{3+} sublattice-mediated energy migration. A depletion efficiency of about 88% for Tb^{3+} emission was obtained by the addition of the 810-nm depletion laser.

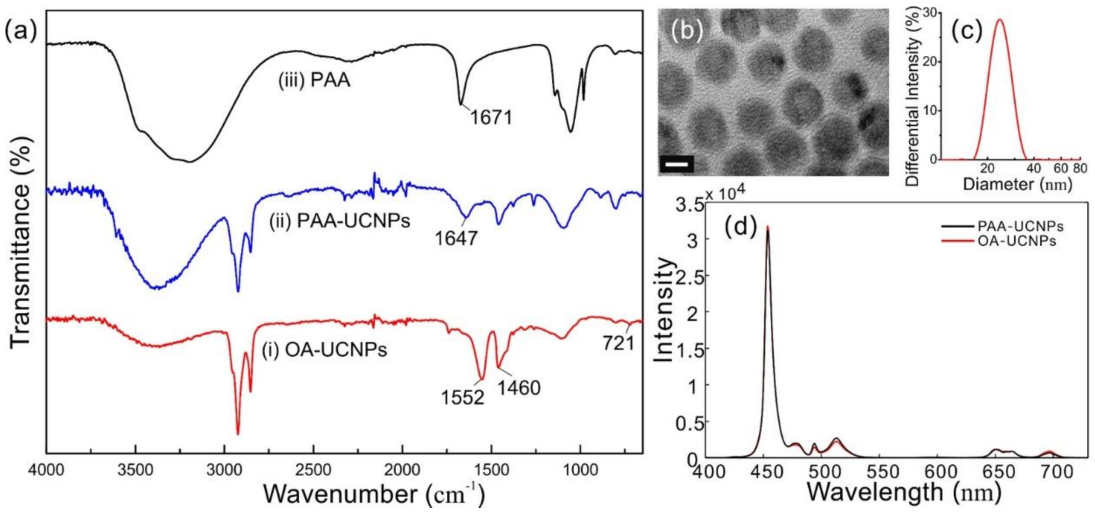

Supplementary Figure 17 | Characterization of COOH-functionalized hydrophilic PAA-UCNPs. (a) FTIR spectra of (i) OA-UCNPs, (ii) PAA-UCNPs and (iii) pure PAA. **(b)** TEM image of PAA-UCNPs (Scale bar: 5 nm). **(c)** Size distribution of PAA-UCNPs measured by DLS. **(d)** Emission spectra and brightness comparison between the OA-UCNPs and the PAA-UCNPs (I_{975}=700 kW cm^{-2}). Concentrated UCNP dispersion was spin-coated onto a glass slide repeatedly to form a uniform film of nanoparticles and then detected under the objective. The bands at 1552 cm^{-1} and 1460 cm^{-1} are associated with the asymmetric (υ_{as}) and symmetric (υ_{s}) stretching vibration of -COO^{-} groups of OA^{-}, since the oleic acid molecules are chemically bonded to the UCNP surface through the coordination between the -COO^{-} group and the RE^{3+} ions[17, 18]. After reaction with PAA, the band at 1552 cm^{-1} corresponding to asymmetric stretching vibration of carboxylate anions became inconspicuous, and the band of PAA measured at 1671 cm^{-1} was shifted to 1647 cm^{-1}, suggesting successful PAA modification on the surfaces of nanoparticles[19, 20]. Furthermore, the peak at 721 cm^{-1}, associated with the in-plane rocking vibration mode of -(CH_2)n- (n＞4) of oleic acid, disappeared in the PAA-UCNPs[21]. Based on these results, it can be inferred that the oleic acids on the surface of UCNPs have been successfully replaced with PAA ligands. The PAA-UCNPs exhibited an average

hydrodynamic diameter of 24 nm, obtained by DLS measurement, which is larger than that observed from TEM measurement (11.8±2.2 nm).

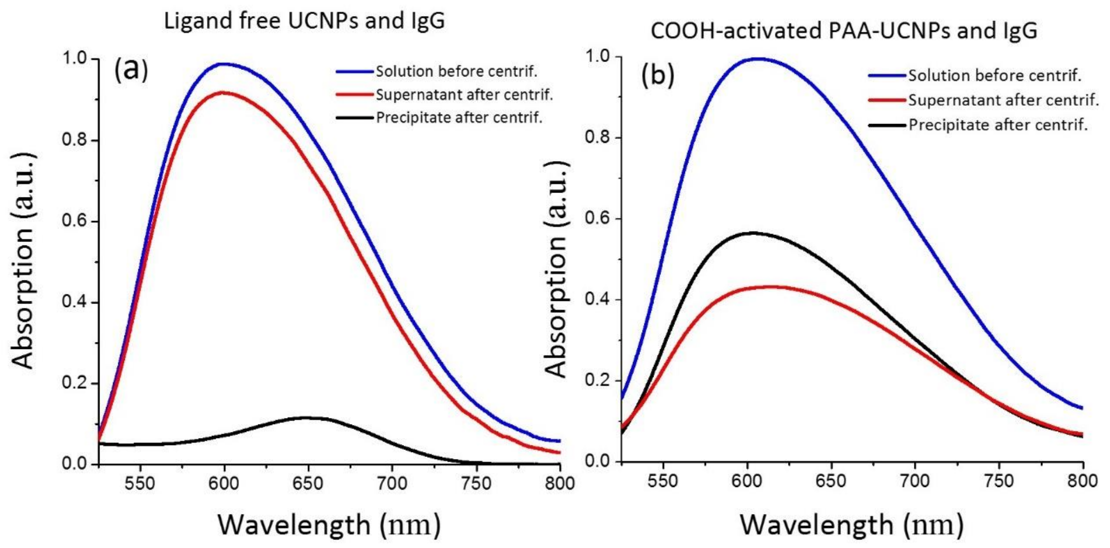

Supplementary Figure 18 | Characterization of bioconjugation of PAA-UCNPs with the secondary antibody IgG. Absorption spectra of the mixtures of the secondary antibody IgG with **(a)** HCl treated ligand-free UCNPs and **(b)** COOH-activated PAA-UCNPs. The samples of the mixture solution before centrifugation (blue solid line), supernatant after centrifugation (red solid line) and the precipitate (after centrifugation) re-dissolved solution (black solid line) were measured using the Bradford protein assay method[22], respectively. As shown in **(a)**, no obvious change was observed in the intensity of the characteristic peak at 595 nm as well as in the profile of the absorption spectrum of IgG solution, indicating that the ligand-free UCNPs did not interact with IgG molecules (no bioconjugation) and thus IgG molecules can hardly be precipitated under this centrifugal condition. The weak absorption centered at 650 nm could be due to rare earth absorption. On the contrary, the significant difference in the spectra shown in **(b)** indicates a successful bioconjugation between the COOH-activated PAA-UCNPs and IgG. The intensity of the characteristic peak for IgG ($I_{595\text{-nm}}$) of the precipitate re-dissolved solution was obviously larger than that of the supernatant after centrifugation, revealing that the majority of IgG molecules were bioconjugated onto the surfaces of the COOH-activated PAA-UCNPs.

Supplementary Table 1 | Measured decay lifetimes of the 455-nm emission of UCNPs with different Tm^{3+} doping concentrations under 975-nm&810-nm simultaneous excitation with varying intensity of 810-nm laser (lifetime in μs).

Power Density (810 nm) \ Concentrations	0.5% Tm^{3+}	2% Tm^{3+}	5% Tm^{3+}	7% Tm^{3+}	10% Tm^{3+}	15% Tm^{3+}	20% Tm^{3+}
0 (980 nm only)	200.5	135.4	90.1	75.7	32.9	13.3	8.1
0.30 MW cm^{-2}	198.2	128.8	85.2	65.2	29.6	11.1	5.8
1.48 MW cm^{-2}	194.6	123.0	73.1	55.3	25.7	8.8	4.1
2.95 MW cm^{-2}	190.2	116.1	69.8	54.6	22.9	7.5	3.3
5.90 MW cm^{-2}	183.3	109.5	66.1	53.8	20.9	6.4	2.7
11.8 MW cm^{-2}	169.3	102.1	64.1	50.3	18.6	5.6	2.3
15.7 MW cm^{-2}	152.0	96.5	61.0	48.2	18.0	5.4	2.2

Supplementary Table 2 | The values of key constants and rate parameters used in the simulations for the 455-nm emission modulation of UCNPs at low and high Tm^{3+} doping levels.

	c_1 ($cm^3\,s^{-1}$)	c_2 ($cm^3\,s^{-1}$)	c_3 ($cm^3\,s^{-1}$)	c_4 ($cm^3\,s^{-1}$)	w_1 ($cm^3\,s^{-1}$)	w_2 ($cm^3\,s^{-1}$)
10%	2.0×10^{-16}[a]	5.3×10^{-16}[a]	5.3×10^{-16}[a]	6.0×10^{-16}[a]	6.0×10^{-17}[b]	3.0×10^{-16}[b]
0.5%	5.0×10^{-19}[a]	1.3×10^{-18}[a]	1.3×10^{-18}[a]	1.5×10^{-18}[a]	1.0×10^{-17}[b]	5.0×10^{-17}[b]

	w_3 ($cm^3\,s^{-1}$)	w_4 ($cm^3\,s^{-1}$)	σ_{d1}^{a} (cm^2)	σ_{d2}^{a} (cm^2)	σ_{d3}^{a} (cm^2)	σ_{d1}^{se} (cm^2)
10%	2.5×10^{-16}[b]	3.0×10^{-16}[b]	9.0×10^{-22}[c]	1.0×10^{-24}[c]	2.0×10^{-23}[c]	3.0×10^{-21}[c]
0.5%	4.2×10^{-17}[b]	5.0×10^{-17}[b]	9.0×10^{-22}[c]	1.0×10^{-24}[c]	2.0×10^{-23}[c]	3.0×10^{-21}[c]

	σ_{d2}^{se} (cm^2)	σ_{d3}^{se} (cm^2)	τ_{Yb1} (s)	τ_1 (s)	τ_3 (s)	τ_4 (s)
10%	5.0×10^{-24}[c]	9.0×10^{-21}[c]	3.3×10^{-4}[d]	1.9×10^{-3}[d]	3.7×10^{-4}[d]	4.7×10^{-4}[d]
0.5%	5.0×10^{-24}[c]	9.0×10^{-21}[c]	5.0×10^{-4}[d]	2.8×10^{-3}[d]	5.6×10^{-4}[d]	7.0×10^{-4}[d]

	τ_5 (s)	τ_6 (s)	τ_7 (s)	τ_8 (s)	β_2 (s^{-1})	β_4 (s^{-1})
10%	8.0×10^{-4}[d]	2.5×10^{-4}[d]	1.5×10^{-4}[d]	3.0×10^{-5}[d]	3.4×10^{4}[e]	1.0×10^{5}[e]
0.5%	1.2×10^{-3}[d]	3.8×10^{-4}[d]	2.3×10^{-4}[d]	4.5×10^{-5}[d]	3.4×10^{4}[e]	1.0×10^{5}[e]

	b_{30}	b_{31}	b_{60}	b_{61}	b_{62}	b_{63}
10%	0.85[f]	0.15[f]	0.65[f]	0.15[f]	0.16[f]	0.14[f]
0.5%	0.85[f]	0.15[f]	0.65[f]	0.15[f]	0.16[f]	0.14[f]

	b_{70}	b_{71}	b_{73}	b_{74}	b_{75}	b_{81}
10%	0.48[f]	0.40[f]	0.03[f]	0.03[f]	0.06[f]	0.58[f]
0.5%	0.48[f]	0.40[f]	0.03[f]	0.03[f]	0.06[f]	0.58[f]

	b_{82}
10%	0.42[f]
0.5%	0.42[f]

[a] Estimated from Ivanova *et al.*[15] and Tkachuk *et al.*[23]

[b] Estimated from Braud *et al.*[24]

[c] Estimated from Peterka *et al.*[25], Medoidze *et al.*[26] and Smith *et al.*[27]

[d] Estimated from Villanueva-Delgado *et al.*[28] and Walsh *et al.*[29]

[e] From Ivanova *et al.*[15]

[f] From Villanueva-Delgado *et al.*[28]

Supplementary References

1. Bradford MM. A rapid and sensitive method for the quantitation of microgram quantities of protein utilizing the principle of protein-dye binding. *Analytical biochemistry* **72**, 248-254 (1976).

2. Willig KI, Harke B, Medda R, Hell SW. STED microscopy with continuous wave beams. *Nature Method* **4**, 915-918 (2007).

3. Smith AV, Smith JJ. Mode instability thresholds for Tm-doped fiber amplifiers pumped at 790 nm. *Opt. Express* **24**, 975-992 (2016).

4. Peterka P, Kasik I, Dhar A, Dussardier B, Blanc W. Theoretical modeling of fiber laser at 810 nm based on thulium-doped silica fibers with enhanced $^3H_4$ level lifetime. *Opt. Express* **19**, 2773-2781 (2011).

5. Medoidze TD, Melikishvili ZG. Ultraviolet and visible emission cross-sections for Tm^{3+} : $YLiF_4$ laser system. *Laser Physics Letters* **1**, 65-68 (2004).

6. Walsh BM, Barnes NP. Comparison of Tm:ZBLAN and Tm: silica fiber lasers; Spectroscopy and tunable pulsed laser operation around 1.9 µm. *Applied Physics B* **78**, 325-333 (2004).

7. Zhang H, Jia T, Shang X, Zhang S, Sun Z, Qiu J. Mechanisms of the blue emission of $NaYF_4$:Tm^{3+} nanoparticles excited by an 800 nm continuous wave laser. *Phys. Chem. Chem. Phys.* **18**, 25905-25914 (2016).

8. Stoneman RC, Esterowitz L. Efficient 1.94-µm Tm:YAlO laser. *IEEE J. Select. Topics Quantum Electron.* **1**, 78-81 (1995).

9. Wen X*, et al.* Highly Tm^{3+} doped germanate glass and its single mode fiber for 2.0 µm laser. *Scientific Reports* **6**, 20344 (2016).

10. Chen X*, et al.* Confining energy migration in upconversion nanoparticles towards deep ultraviolet lasing. *Nature Communications* **7**, 10304 (2016).

11. Gruber JB, Krupke WF, Poindexter JM. Crystal-Field Splitting of Trivalent Thulium and Erbium J Levels in Yttrium Oxide. *Journal of Chemical Physics* **41**, 3363-3377 (1964).

12. Antonov VA, Arsenev PA, Bienert KE, Potemkin AV. Spectral properties of rare-earth ions in $YAlO_3$ crystals. *Physica Status Solidi* **19**, 289-299 (1973).

13. Liu Y*, et al.* Amplified stimulated emission in upconversion nanoparticles for super-resolution nanoscopy. *Nature* **543**, 229-233 (2017).

14. Zhao J*, et al.* Single-nanocrystal sensitivity achieved by enhanced upconversion luminescence. *Nature Nanotechnology* **8**, 729 (2013).

15. Ivanova SE, Tkachuk AM, Mirzaeva A, Pellé F. Spectroscopic study of thulium-activated double sodium yttrium fluoride $Na_{0.4}Y_{0.6}F_{2.2}$:Tm^{3+} crystals:I.

Intensity of spectra and luminescence kinetics. *Optics & Spectroscopy* **105**, 228-241 (2008).

16. Wang F*, et al.* Tuning upconversion through energy migration in core–shell nanoparticles. *Nature Materials* **10**, 968-973 (2011).

17. Bogdan N, Vetrone F, Ozin GA, Capobianco JA. Synthesis of ligand-free colloidally stable water dispersible brightly luminescent lanthanide-doped upconverting nanoparticles. *Nano Letters* **11**, 835 (2011).

18. Zhang T, Ge J, Yongxing Hu A, Yin Y. A General Approach for Transferring Hydrophobic Nanocrystals into Water. *Nano Letters* **7**, 3203 (2007).

19. Jia X*, et al.* Polyacrylic acid modified upconversion nanoparticles for simultaneous pH-triggered drug delivery and release imaging. *Journal of Biomedical Nanotechnology* **9**, 2063 (2013).

20. Zhao P*, et al.* Upconversion fluorescent strip sensor for rapid determination of Vibrio anguillarum. *Nanoscale* **6**, 3804-3809 (2014).

21. Skoog DA, West DM. *Principles of Instrumental Analysis*, Second edn. Saunders College (1980).

22. Pierce J, Suelter C. An evaluation of the Coomassie brilliant blue G-250 dye-binding method for quantitative protein determination. *Analytical biochemistry* **81**, 478-480 (1977).

23. Tkachuk AM, Razumova IK, Perlin EY, Joubert MF, Moncorge R. Luminescence self-quenching in Tm^{3+}:YLF crystals: II. The luminescence decay and macrorates of energy transfer. *Optics & Spectroscopy* **90**, 78-88 (2001).

24. Braud A, Girard S, Doualan JL, Thuau M, Moncorgé R, Tkachuk AM. Energy-transfer processes in Yb:Tm-doped KY_3F_{10}, $LiYF_4$, and BaY_2F_8 single crystals for laser operation at 1.5 and 2.3 μm. *Physical Review B* **61**, 5280 (2000).

25. Peterka P, Kasik I, Dhar A, Dussardier B, Blanc W. Theoretical modeling of fiber laser at 810 nm based on thulium-doped silica fibers with enhanced $^3H_4$ level lifetime. *Opt. Express* **19**, 2773 (2011).

26. Medoidze TD, Melikishvili ZG. Ultraviolet and visible emission cross-sections for Tm^{3+}: $YLiF_4$ laser system. *Laser Physics Letters* **1**, 65–68 (2010).

27. Smith AV, Smith JJ. Mode instability thresholds for Tm-doped fiber amplifiers pumped at 790 nm. *Opt. Express* **24**, 975 (2015).

28. Villanueva-Delgado P, Biner D, Krämer KW. Judd-Ofelt analysis of β-$NaGdF_4$: Yb^{3+}, Tm^{3+} and β-$NaGdF_4$:Er^{3+} single crystals. *Journal of Luminescence* **189**, 84-90 (2016).

29. Walsh BM, Barnes NP. Comparison of Tm: ZBLAN and Tm: silica fiber lasers; Spectroscopy and tunable pulsed laser operation around 1.9 µm. *Applied Physics B* **78**, 325-333 (2004).
